# Safety outcomes in patients with rheumatoid arthritis treated with abatacept: results from a multinational surveillance study across seven European registries

**DOI:** 10.1186/s13075-023-03067-x

**Published:** 2023-06-12

**Authors:** Alyssa Dominique, Merete Lund Hetland, Axel Finckh, Jacques-Eric Gottenberg, Florenzo Iannone, Roberto Caporali, Tzuyung Douglas Kou, Dan Nordstrom, Maria Victoria Hernandez, Carlos Sánchez-Piedra, Fernando Sánchez-Alonso, Karel Pavelka, T. Christopher Bond, Teresa A. Simon

**Affiliations:** 1 Bristol Myers Squibb, Princeton, NJ 08534 USA; 2 DANBIO and Copenhagen Center for Arthritis Research (COPECARE), Center for Rheumatology and Spine Diseases, Centre of Head and Orthopedics, Rigshospitalet, Glostrup, Denmark; 3 Department of Clinical Medicine, Faculty of Health and Medical Sciences, University of Copenhagen, Copenhagen, Denmark; 4 Division of Rheumatology, University Hospitals of Geneva, Geneva, Switzerland; 5 Strasbourg University Hospitals, Strasbourg, France; 6 DETO-Rheumatology Unit, University of Bari, Bari, Italy; 7 Department of Clinical Sciences and Community Health, University of Milan, Milan, Italy; 8 ASST PINI-CTO Hospital, Milan, Italy; 9 Departments of Medicine and Rheumatology, Helsinki University Hospital and Helsinki University, Helsinki, Finland; 10 Rheumatology Department, Hospital Clinic of Barcelona, Barcelona, Spain; 11 BIOBADASER, Research Unit, Sociedad Española de Reumatología, Madrid, Spain; 12 Institute of Rheumatology, Prague, Czech Republic

**Keywords:** Rheumatoid arthritis, Abatacept, Biologic DMARD, Safety, Infections, Malignancy

## Abstract

**Background:**

Patients with rheumatoid arthritis (RA) have an increased risk of infection and malignancy compared with the general population. Infection risk is increased further with the use of disease-modifying antirheumatic drugs (DMARDs), whereas evidence on whether the use of biologic DMARDs increases cancer risk remains equivocal. This single-arm, post-marketing study estimated the incidence of prespecified infection and malignancy outcomes in patients with RA treated with intravenous or subcutaneous abatacept.

**Methods:**

Data were included from seven European RA quality registries: ATTRA (Anti-TNF Therapy in Rheumatoid Arthritis [Czech Republic]), DANBIO (Danish Rheumatologic Database), ROB-FIN (National Registry of Antirheumatic and Biological Treatment in Finland), ORA (Orencia and Rheumatoid Arthritis [France]), GISEA (Italian Group for the Study of Early Arthritis), BIOBADASER (Spanish Register of Adverse Events of Biological Therapies in Rheumatic Diseases), and the SCQM (Swiss Clinical Quality Management) system. Each registry is unique with respect to design, data collection, definition of the study cohort, reporting, and validation of outcomes. In general, registries defined the index date as the first day of abatacept treatment and reported data for infections requiring hospitalization and overall malignancies; data for other infection and malignancy outcomes were not available for every cohort. Abatacept exposure was measured in patient-years (p-y). Incidence rates (IRs) were calculated as the number of events per 1000 p-y of follow-up with 95% confidence intervals.

**Results:**

Over 5000 patients with RA treated with abatacept were included. Most patients (78–85%) were female, and the mean age range was 52–58 years. Baseline characteristics were largely consistent across registries. Among patients treated with abatacept, IRs for infections requiring hospitalization across the registries ranged from 4 to 100 events per 1000 p-y, while IRs for overall malignancy ranged from 3 to 19 per 1000 p-y.

**Conclusions:**

Despite heterogeneity between registries in terms of design, data collection, and ascertainment of safety outcomes, as well as the possibility of under-reporting of adverse events in observational studies, the safety profile of abatacept reported here was largely consistent with previous findings in patients with RA treated with abatacept, with no new or increased risks of infection or malignancy.

**Supplementary Information:**

The online version contains supplementary material available at 10.1186/s13075-023-03067-x.

## Introduction

Compared with the general population, patients with rheumatoid arthritis (RA) are at an increased risk of certain events, specifically infections and malignancies [1–4]. Patients with RA are at a modestly increased risk of malignancy overall—for lung cancer and lymphoma, in particular—but at a decreased risk of colorectal and breast cancers [3, 4]. Data suggest that the risk of infections in patients with RA is further increased with the use of conventional synthetic (cs) disease-modifying antirheumatic drugs (DMARDs) and biologic (b)/targeted synthetic (ts) DMARDs [1, 2]. Although there has been no finding that the risk of malignancies with the use of b/tsDMARDs is increased in the RA population, evidence regarding long-term safety continues to emerge [2, 5–14].

The bDMARD abatacept, a selective T cell co-stimulation modulator that blocks the interaction between CD80/CD86 on antigen-presenting cells and CD28 on T cells [15], is approved for the treatment of RA [16, 17]. Abatacept is effective in reducing disease activity, preventing or slowing radiologic disease progression, and improving physical function and health-related quality of life in adult patients with early [18, 19] and established [15, 20–25] RA. Data from randomized, controlled clinical trials have shown abatacept to be well tolerated, with adverse event rates similar to those of placebo, and to have a consistent and favourable safety profile over the longer term [15, 26–28]. A recently published, integrated safety analysis for abatacept included data from nine clinical trials in adult patients with RA treated with abatacept (*n* = 2653) or placebo (*n* = 1485) for a mean (standard deviation) duration of exposure of 10.8 (3.3) and 10.3 (3.5) months, respectively [29]. The study showed that the incidence rates (IRs) for overall infections, serious infections/infestations, opportunistic infections, and malignancies were similar between the abatacept and placebo groups.

An abatacept global post-marketing epidemiology programme was initiated in 2006 in North America and 2008 in Europe to monitor the safety, specifically infections and malignancies, of abatacept using real-world data sources. The programme included observational data from a geographically diverse group of patients and clinical practices gathered in bDMARD or disease registries in Europe and North America, as well as administrative healthcare claims databases from North America. A retrospective, observational study using administrative data from three large US healthcare databases showed a slightly but statistically significantly increased risk in total malignancies but no increased risk for specific cancers (lung, breast, lymphoma), infections requiring hospitalization, or opportunistic infections with abatacept versus b/tsDMARDs [30]. In a cohort study, the use of abatacept was associated with an increased incidence of cancer overall and a significantly increased incidence of non-melanoma skin cancer compared with other bDMARDs [11]. Additionally, a recent large pooled analysis of randomized trials demonstrated no increased risk of infections with abatacept compared with non-bDMARDs [31]. An observational study using VigiBase, the World Health Organization’s global database of individual case safety reports, showed that exposure to abatacept (compared with other bDMARDs) in patients with RA was significantly associated with an increased risk of reporting melanoma [32]. In a register-based prospective cohort study of patients with RA, abatacept was associated with an increased risk of developing squamous cell skin cancer compared with TNFi [7].

The present study was designed to complement the abatacept global post-marketing epidemiology programme, comply with the European regulatory authority requirements, and contribute to the body of evidence on the safety of abatacept. The study used data from seven European-based RA registries to estimate the incidence of prespecified outcomes, namely infections and malignancies, in patients with RA treated with abatacept.

## Methods

### Study design and data sources

This single-arm study included data from patients treated with intravenous or subcutaneous abatacept taken from seven established longitudinal observational RA registries based in Europe: ATTRA (Anti-TNF Therapy in Rheumatoid Arthritis [Czech Republic]), DANBIO (Danish Rheumatologic Database), ROB-FIN (National Registry of Antirheumatic and Biological Treatment in Finland), ORA (Orencia and Rheumatoid Arthritis [France]), GISEA (Italian Group for the Study of Early Arthritis), BIOBADASER (Spanish Register of Adverse Events of Biological Therapies in Rheumatic Diseases), and the SCQM (Swiss Clinical Quality Management) system.

The study was conducted initially using data from ATTRA, DANBIO, ROB-FIN, and SCQM, with the addition of three registries—ORA, GISEA, and BIOBADASER—in 2014. Each data source is unique with respect to the design, data collection, reporting, and validation of outcomes. The characteristics of each registry are shown in Table 1.

**Table 1:** Characteristics of registries used for identifying patients with RA in the post-marketing epidemiology abatacept study

	**ATTRA**	**DANBIO **[33, 34]	**ROB-FIN**	**ORA**	**GISEA**	**BIOBADASER**	**SCQM**
Country	Czech Republic	Denmark	Finland	France	Italy	Spain	Switzerland
Year of registry initiation	2002	2000	1999	2007	2008	2007	1996
Register design	National register	National register	National register	Multicentre registry^a^	Multicentre registry of bDMARDs	National register	National register
Age (years)	≥ 18	≥ 18	≥ 18	≥ 18	≥ 18	≥ 18	≥ 16
Data availability	8 August 2007 to 27 November 2017	15 June 2007 to 3 December 2017^b^	September 2007 to 31 December 2015	June 2007 to 3 June 2018	Through March 2014 (outcomes data) and through November 2015 (baseline data)	2007 to 15 November 2017^c^	Follow-up 1 January 2009 to 30 June 2017 (infections requiring hospitalization) or 1 October 2007 to 30 June 2017 (tuberculosis, malignancies)
Infection outcomes							
Hospitalized infections	Yes	Yes	Yes	Yes	Yes^d^	Yes	Yes
Opportunistic infections	Yes	Yes	NR	NR	NR	Yes	NR
Tuberculosis	Yes	Yes	Yes	NR	NR	Yes	Yes
Malignancy outcomes							
Overall malignancy	Yes	Yes	Yes	Yes	Yes^d^	Yes	Yes
Breast Cancer	Yes	Yes	Yes	Yes^e^	NR	Yes	Yes
Lung cancer	Yes	Yes	Yes	Yes^e^	NR	Yes	Yes
Lymphoma	Yes	Yes	Yes	Yes^e^	NR	Yes	Yes
Confirmation of infections^f^	MedDRA	ICD-10 codes	ICD-10 codes	Patient adverse event summary narratives	ICD codes	MedDRA	Identified by the treating rheumatologist
Confirmation of malignancy	MedDRA	ICD-10 codes	Linkage to national hospitalization discharge register (HILMO); ICD codes^g^	Patient adverse event summary narratives	ICD-codes based on the reports of pathologists	MedDRA	Identified by the treating rheumatologist^e^

*ATTRA* Anti-TNF Therapy in Rheumatoid Arthritis, *bDMARD* biologic disease-modifying antirheumatic drug, *BIOBADASER* Spanish Register of Adverse Events of Biological Therapies in Rheumatic Diseases, *DANBIO* Danish Rheumatologic Database, *GISEA* Italian Group for the Study of Early Arthritis, *HILMO* Finnish nationwide social and healthcare data collection and reporting system, *ICD* International Classification of Diseases, *MedDRA* Medical Dictionary for Drug Regulatory Activities, *NR* not reported, *ORA* Orencia and Rheumatoid Arthritis, *RA* rheumatoid arthritis, *ROB-FIN* National Registry of Biological Treatment in Finland, *SCQM* Swiss Clinical Quality Management

^a^Established by the French Society of Rheumatology to investigate the long-term safety and effectiveness of intravenous abatacept in patients with RA

^b^For DANBIO, the study period corresponds to the time period when abatacept became available in Denmark; patients were also required to have follow-up data from the Danish National Patient Registry for the final report

^c^BIOBADASER 2.0 and 3.0

^d^Reported as adverse event counts

^e^Reported in patient adverse event summary narratives

^f^For infections resulting in hospitalization, the infection code was the first code indicating the primary reason for hospitalization. In DANBIO, both primary and secondary codes were included as patients with RA may have had RA as the primary code

^g^If the description of the malignancy or the infection was not clear, an SCQM data analyst or study nurse could request additional information on the specific type or location of the malignancy

This study was conducted in accordance with the International Society for Pharmacoepidemiology Guidelines for Good Pharmacoepidemiology Practices, applicable regulatory requirements, and the principles of the Declaration of Helsinki.

### Patient population

Patients diagnosed with RA who had a record of treatment with abatacept and follow-up information available were eligible for inclusion in the study. The definition of the study cohort for each registry is included in Table 1. Patient identification, eligibility criteria, and definition of study cohorts that qualified a patient for inclusion varied between the individual registries.

### Outcomes

Each registry was unique with respect to the outcomes reported; thus, not all outcomes are available for every cohort (Table 1). All seven registries reported data for infections requiring hospitalization and overall malignancies. For the GISEA registry, infections requiring hospitalization and malignancies were reported as adverse event counts. For specific infection outcomes, opportunistic infections were reported only for the ATTRA, DANBIO, and BIOBADASER registries; tuberculosis was reported by all registries apart from ORA and GISEA. For specific malignancy outcomes, lymphoma, breast, and lung cancers were reported by all registries apart from GISEA. For the ORA registry, the occurrence of breast cancer, lung cancer, and lymphoma was reported in the patient adverse event summary narratives. The exclusion criteria for patients with a history of cancer prior to abatacept exposure were not standardized in this study; however, in the DANBIO registry, patients who had previous diagnoses of cancer (except non-melanoma skin cancer) were excluded from the IR calculations.

### Exposure

The calculation of abatacept exposure was computed by the individual registries. In general, the index date was the first day of treatment with abatacept and exposure was measured in patient-years (p-y).

For infections, patient time was calculated from the index date until the event of interest, the end of the newly initiated treatment plus 90-day lag time, death, emigration (DANBIO), the end of the study, or the last follow-up date (if earlier than the 90-day lag time in the ORA registry), whichever occurred first.

As malignancies often have latency periods, the risk associated with a specific drug is expected to continue beyond the immediate period of exposure to any treatment. Thus, patient time of exposure to abatacept for malignancies was extended to the end of the follow-up period regardless of whether the patient discontinued the drug. In addition, different registries calculated exposure time for malignancy slightly differently (Additional file 1).

### Statistical analyses

Within each registry, baseline demographics and clinical characteristics were computed with descriptive statistics for categorical and continuous variables.

There was some variation in the methodology for the calculation of IRs for each registry (Additional file 1). IRs for each prespecified outcome were provided by the individual registries as the number of events per 1000 total p-y of follow-up with 95% confidence intervals (CIs), except for all IRs reported for GISEA and for some malignancy IRs reported for ORA. For GISEA, IRs were calculated using the mean duration of therapy (in months) and adverse event counts for infections requiring hospitalization and overall malignancies. For ORA, IRs for breast cancer, lung cancer, and lymphoma were based on patient narrative data and p-y provided; follow-up for incidence of cancers is the overall follow-up period. CIs were not provided by the ORA or GISEA registries for any of the IRs.

## Results

### Patient demographics and clinical characteristics

Over 5000 patients with RA treated with abatacept were included in this study (Table 2). Most patients (78–85%) were female, and the mean age ranged from 52.1 to 58.0 years across all registries. Baseline characteristics were largely consistent across the seven different registries, apart from a longer disease duration in the ORA registry. The mean duration of RA ranged from 8.1 to 14.0 years across the seven registries.

**Table 2:** Baseline demographics and clinical characteristics of patients with RA in the post-marketing epidemiology abatacept study

	**ATTRA**	**DANBIO**	**ROB-FIN**	**ORA**	**GISEA**	**BIOBADASER**	**SCQM**^**a**^
Number of patients	335	1213	362	976	433 with 770^b^ total reported events	350	959 infection cohort 1053 malignancy cohort
Patient-years of exposure	862	4513	530	6119	NR	NR	1902 infection cohort 3683 malignancy cohort
Age (years), mean (SD)	52.1 (11.4)	57.2 (12.7)	54.7 (13.5)	58.0 (14.0)	First-line: 58.1 (NR) Second-line: 58.5 (NR) Other lines: 57.5 (NR)	57.5 (13.5)	58 (13)
Female, *n* (%)	270 (81)	941 (78)	308 (85)	771 (79)	654 (85)	276 (79)	~ 79%
Duration of RA (years), mean (SD)	8.1 (7.4)	10.1 (9.9)	12.8 (11.0)	14.0 (10.0)	First-line: 9.2 (NR) Second-line: 11.3 (NR) Other lines: 13.3 (NR)	NR	~ 11 (NR)

*ATTRA* Anti-TNF Therapy in Rheumatoid Arthritis, *BIOBADASER* Spanish Register of Adverse Events of Biological Therapies in Rheumatic Diseases, *DANBIO* Danish Rheumatologic Database, *GISEA* Italian Group for the Study of Early Arthritis, *NR* not reported, *ORA* Orencia and Rheumatoid Arthritis, *RA* rheumatoid arthritis, *ROB-FIN* National Registry of Biological Treatment in Finland, *SCQM* Swiss Clinical Quality Management, *SD* standard deviation

^a^Patient populations differed for the different outcomes; baseline characteristics of the patient populations for each outcome were similar

^b^Total for first-line, second-line, and other lines of treatment from 2007 to November 2015

### Infections

**Fig. 1:**
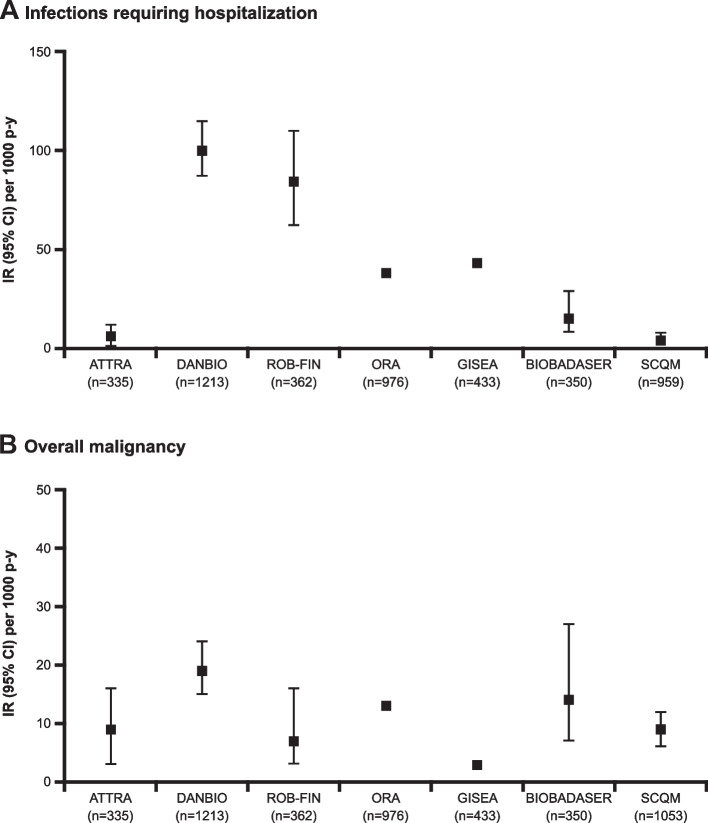
Incidence rates/1000 patient-years (95% CI) for **A** infections requiring hospitalization and **B** overall malignancy. For GISEA, IRs were calculated using the mean duration of therapy (in months) and adverse event counts for infections requiring hospitalization and overall malignancies; for overall malignancy, the calculated IR used first- and second-line abatacept adverse event counts from November 2015. No CIs were provided by the ORA or GISEA registries for any of the IRs. For SCQM, IRs are by exposure group with mid-imputed dates of occurrence. ATTRA, Anti-TNF Therapy in Rheumatoid Arthritis; BIOBADASER, Spanish Register of Adverse Events of Biological Therapies in Rheumatic Diseases; DANBIO, Danish Rheumatologic Database; CI, confidence interval; GISEA, Italian Group for the Study of Early Arthritis; IR, incidence rate; ORA, Orencia and Rheumatoid Arthritis; p-y, patient-years; ROB-FIN, National Registry of Antirheumatic and Biological Treatment in Finland; SCQM, Swiss Clinical Quality Management

Among patients treated with abatacept, the IRs for infections requiring hospitalization ranged from 4 to 100 per 1000 p-y (Table 3, Fig. 1A). The highest rates were observed in the DANBIO registry, with an IR of 100 per 1000 p-y (95% CI: 87, 115), corresponding to 196 events in 1213 patients with 1955 p-y of exposure. The lowest IRs were observed in the SCQM (4 per 1000 p-y [95% CI: 2, 8]) and ATTRA (6 per 1000 p-y [95% CI: 0, 12]) registries.

**Table 3:** Incidence rates/1000 patient-years (95% CI), number of events, and patient-years of exposure for infections

	**ATTRA (*n* = 335)**	**DANBIO (*n* = 1213)**	**ROB-FIN (*n* = 362)**	**ORA**^**a**^** (*n* = 976)**	**GISEA (*n* = 433)**	**BIOBADASER (*n* = 350)**	**SCQM**^**b**^** (*n* = 959/974)**
Infections requiring hospitalization							
IR per 1000 p-y (95% CI)	6 (0, 12)	100 (87, 115)	84 (62, 110)	38^c^ (NR)	43^d^ (NR)	15 (8, 29)	4 (2, 8)
Number of events (p-y)	5 (NR)	196 (1955)	49 (NR)	298 (NR)	NR (NR)	9 (578)	7 (1902)
Opportunistic infections							
IR per 1000 p-y (95% CI)	0	1 (0, 3)	NR	NR^d^	NR	14 (7, 27)	NR
Number of events (p-y)	0 (0)	2 (2233)	NR (NR)	NR (NR)	NR (NR)	8 (578)	NR (NR)
Tuberculosis							
IR per 1000 p-y (95% CI)	6 (0, 12)	1 (0, 3)	0 (0, 6)	NR	NR	0	1.5 (1, 5)
Number of events (p-y)	5 (NR)	2 (2230)	0 (0)	NR (NR)	NR (NR)	0 (0)	3 (1941)

*ATTRA* Anti-TNF Therapy in Rheumatoid Arthritis, *BIOBADASER* Spanish Register of Adverse Events of Biological Therapies in Rheumatic Diseases, *CI* confidence interval, *DANBIO* Danish Rheumatologic Database, *GISEA* Italian Group for the Study of Early Arthritis, *IR* incidence rate, *NR* not reported, *ORA* Orencia and Rheumatoid Arthritis, *p-y* patient-years, *ROB-FIN* National Registry of Biological Treatment in Finland, *SCQM* Swiss Clinical Quality Management

^a^No CIs were provided in the report submitted by the French Society of Rheumatology to health authorities

^b^The sample size for infections requiring hospitalization was 959; for tuberculosis, the sample size was 974. IRs are by exposure group with mid-imputed dates of occurrence

^c^Intravenous antibiotic therapy and/or hospitalization

^d^Calculated IR from the final study report from GISEA

^d^For ORA, NR means not reported in the report submitted to the French Health Authorities by the French Society of Rheumatology

The IRs for opportunistic infections ranged from 0 to 14 per 1000 p-y (Table 3). In BIOBADASER, the IR was 14 per 1000 p-y (95% CI: 7, 27), corresponding to 8 events in 350 patients with 578 p-y of exposure. In DANBIO, there were two cases of opportunistic infections occurring in 1213 patients with 2233 p-y of exposure. There were no occurrences of opportunistic infections reported in the ATTRA registry.

The IRs for tuberculosis ranged from 0 to 6 per 1000 p-y with the number of events ranging from 0 in the ROB-FIN and BIOBADASER registries to 5 in the ATTRA registry (Table 3).

### Malignancies

The IRs for overall malignancies in patients treated with abatacept were low and ranged from 3 to 19 per 1000 p-y (Table 4, Fig. 1B). The highest rates were observed in the DANBIO registry with an IR of 19 per 1000 p-y (95% CI: 15, 24), corresponding to 70 events in 1213 patients with 3642 p-y of exposure.

**Table 4:** Incidence rates/1000 patient-years (95% CI), number of events, and patient-years of exposure for malignancies

	**ATTRA (*n* = 335)**	**DANBIO (*n* = 1213)**	**ROB-FIN (*n* = 362)**	**ORA (*n* = 976)**	**GISEA (*n* = 433)**	**BIOBADASER (*n* = 350)**	**SCQM**^**a**^** (*n* = 1053)**
Overall malignancy							
IR per 1000 p-y (95% CI)	9 (3, 16)	19 (15, 24)	7 (3, 16)	13 (NR)	3^b^ (NR)	14 (7, 27)	9 (6, 12)
Number of events (p-y)	8 (NR)	70 (3642)	6 (NR)	92 (NR)	NR (NR)	8 (578)	32 (3683)
Breast							
IR per 1000 p-y (95% CI)	2 (0, 9)	2 (1, 4)	2 (0, 9)	NR (NR)	NR	1.7 (0, 12)	NR
Number of events (p-y)	2 (NR)	6 (2939)	2 (NR)	NR	NR (NR)	1 (578)	NR (NR)
Lung							
IR per 1000 p-y (95% CI)	1 (0, 8)	2 (1, 4)	1.2 (0, 7)	NR (NR)	NR	2 (0, 12)	NR
Number of events (p-y)	1 (NR)	8 (3775)	1 (NR)	NR	NR	1 (578)	NR
Lymphoma							
IR per 1000 p-y (95% CI)	1 (0, 8)	1 (0, 2)	0 (0, 5)	0.7 (NR)	NR	0	NR
Number of events (p-y)	1 (NR)	3 (3779)	0 (NR)	5 (NR)	NR (NR)	0 (578)	NR (NR)

*ATTRA* Anti-TNF Therapy in Rheumatoid Arthritis, *BIOBADASER* Spanish Register of Adverse Events of Biological Therapies in Rheumatic Diseases, *CI* confidence interval, *DANBIO* Danish Rheumatologic Database, *GISEA* Italian Group for the Study of Early Arthritis, *IR* incidence rate, *NR* not reported, *ORA* Orencia and Rheumatoid Arthritis, *p-y* patient-years, *ROB-FIN* National Registry of Biological Treatment in Finland, *SCQM* Swiss Clinical Quality Management

^a^IR is by exposure group with mid-imputed dates of occurrence

^b^Calculated IR using first- and second-line abatacept counts from November 2015

The IRs for breast cancer ranged from 1.7 to 2 per 1000 p-y (Table 4). The IRs for lung cancer and lymphoma were low and ranged from 1 to 2 and 0 to 1 per 1000 p-y, respectively (Table 4).

## Discussion

This real-world observational study of over 5000 patients with RA treated with abatacept showed a range in rates of infection and malignancy events across the included registries. No new or increased risks of infection or malignancy with abatacept were identified. These findings are consistent with the safety profile of abatacept reported in adult patients with active RA in multiple randomized controlled clinical trials, large international observational trials, and several extensive real-world data sources from the literature [29–31, 35].

The IRs of infections requiring hospitalization observed with abatacept in the present study ranged from 4 to 100 per 1000 p-y. A recent study from the abatacept global post-marketing epidemiology programme included observational data from five large international data sources including a total of 6450 patients treated with abatacept, reported the following IRs of infections requiring hospitalization for patients with RA treated with abatacept, csDMARDs, or b/tsDMARDs: 16–56/1000 p-y, 19–46/1000 p-y, and 18–40/1000 p-y, respectively [31]. Compared with these data, the IRs for infections requiring hospitalization in the present study were lower in the ATTRA (6/1000 p-y) and SCQM (4/1000 p-y) registries but higher in the DANBIO (100/1000 p-y) and ROB-FIN (84/1000 p-y) registries. Methodologic differences across the seven registries in this study included the determination of index date, data collection methods, definition of outcomes, and reporting of adverse events. The differences in methodology may have contributed to the wide range of IRs observed. Overall, with the exception of DANBIO, the IRs for infections requiring hospitalization reported in the present study were consistent with reference ranges published previously from the abatacept global post-marketing epidemiology programme [31]. In DANBIO, the higher rates of infections requiring hospitalization may be explained by linkage to the Danish National Patient Registry, which represents a nearly complete Danish population and, therefore, ensured that a high rate of adverse events was captured. Another observational cohort study (2010–2015), which included data from DANBIO and the Anti-Rheumatic Treatment in Sweden Register/Swedish Rheumatology Quality Register, investigated the risk of serious (hospitalized) infections in patients with RA who started treatment with non-tumour necrosis factor (TNF) inhibitor bDMARDs [36]. Over the first 12 months, crude IRs for serious infections ranged from 61 to 70/1000 p-y for abatacept, 71 to 88/1000 p-y for rituximab, and 46 to 61/1000 p-y for tocilizumab, with the Danish rates being consistently higher than the Swedish rates [36].

For other infection outcomes reported in the present study, the IRs for opportunistic infections were low in ATTRA (0/1000 p-y) and DANBIO (1/1000 p-y) and consistent with recently published data for abatacept (0.4–7.8/1000 p-y), csDMARDs (0.3–4.3/1000 p-y), and b/tsDMARDs (0.5–3.8/1000 p-y) [31] but were higher in BIOBADASER (14/1000 p-y). Additionally, IRs of tuberculosis in this study were low (0–1.5/1000 p-y) across four of the five registries that reported data but were higher (IR: 6/1000 p-y) in the ATTRA database. These findings may, in part, be reflective of the variability in the incidence of tuberculosis across Europe [37]. Data from the abatacept global post-marketing epidemiology programme reported IRs of tuberculosis per 1000 p-y of 0.0–8.4, 0.0–6.0, and 0.0–6.3 in patients receiving treatment with abatacept, csDMARDs, and b/tsDMARDs, respectively (data on file). Data from the British Society for Rheumatology Biologics Register (BSRBR), a national prospective observational study comparing rates of tuberculosis in more than 10,000 patients treated with a TNF inhibitor, reported crude IRs of ~ 1.4/1000 p-y [38], while a retrospective cohort study from Taiwan (*n* = 951) reported IRs of tuberculosis of 0.9 and 1.1 per 1000 p-y in patients treated with etanercept and adalimumab, respectively [33]. Another larger retrospective study using data from Taiwan’s National Health Insurance Database reported rates of tuberculosis of 0.55/1000 p-y in 7888 patients with RA treated with a tsDMARD, 1.5/1000 p-y in 3459 patients treated with a bDMARD, and rates of 1.2/1000 p-y for etanercept and 2.9/1000 p-y for adalimumab specifically [34]. Overall, the data reported here are consistent with previously reported data for abatacept, csDMARDs, and other b/tsDMARDs [31, 33, 34, 38].

Similar to the results observed for infections, there was a wide variability in the incidence of overall malignancies across the seven registries included in the present study. Again, this variability may be explained by methodologic differences between the different data sources. Furthermore, although the IR of overall malignancy reported in the DANBIO registry was higher than expected, despite the vast methodologic differences, the IRs for the remaining six registries were similar to those from a recent observational study from the abatacept global post-marketing epidemiology programme that reported IRs/1000 p-y for overall malignancy of 8–11 for abatacept, 9–13 for csDMARDs, and 5–12 for b/tsDMARDs [35]. Data from the present study are also consistent with data reported in an integrated analysis of data from nine randomized, controlled clinical trials, where IRs of malignancy were 13/1000 p-y for patients treated with abatacept versus 14/1000 p-y for patients receiving placebo [29]. Other studies have reported variability in the incidence of overall malignancy with different therapeutic agents used in the treatment of patients with RA. A 2016 study from the BSRBR reported rates of incident malignancy of 33.3 events/1000 p-y for patients treated with a TNF inhibitor, 24.7/1000 p-y for rituximab, and 53.8/1000 p-y for those treated with a non-bDMARD [39]. Other observational studies also reported an increased risk of total malignancies [30] and skin cancers [7, 32] in patients with RA exposed to abatacept. Recently, another Swedish observational study noted a potential increased overall cancer risk in patients with RA treated for 2–5 years with abatacept (IR of 13.6 1000 p-y compared to the general population) [12], but the models presented in the study did not account for the number of prior biologics. There is evidence showing that patients with RA who received abatacept have been exposed to, on average, more than 2 biologic treatments [unpublished data]; this cumulative exposure to biologics may result in an increase in cancer risk [unpublished data].

In contrast, other studies have reported much lower rates of overall malignancy, including IRs of 5.4/1000 p-y observed with bDMARDs versus 7.4/1000 p-y with non-bDMARD treatment in the Taiwan National Health Insurance Research Database [40] and 8.1/1000 p-y with TNF inhibitor therapy and 11.7/1000 p-y with non-bDMARDs (including methotrexate) in another study from the BSRBR [8].

In terms of specific types of cancer, the IRs reported here for breast cancer (1.7–2/1000 p-y), lung cancer (1–2/1000 p-y), and lymphoma (0–1/1000 p-y) are within the expected ranges for abatacept based on previously reported data from the abatacept global post-marketing epidemiology programme (IRs/1000 p-y were 0–4.4 for breast cancer, 0.1–2.8 for lung cancer, and 0–1.1 for lymphoma) [35]. This previous study, which included data from over 110,000 patients across geographically diverse data sources, demonstrated similar rates of breast cancer, lung cancer, and lymphoma among patients treated with abatacept, csDMARDs, and other b/tsDMARDs [35]. It is important to note, however, that issues of channelling bias, differences in methodology between studies, and regional differences in healthcare systems, such as cancer screening methods and surveillance programmes, make direct comparisons of malignancy rates between DMARD cohorts challenging.

The data reported here contribute to the existing body of evidence on the safety of abatacept from randomized controlled trials and the post-marketing epidemiology abatacept programme. The strength of the present study lies in its provision of additional information on the safety of abatacept in over 5000 patients with RA from multiple databases across Europe. A geographically diverse group of patients and clinical practices are represented, with data reported for about 10 years for most of the registries included in the analysis. Baseline characteristics were similar across databases, confirming the validity of combining data. Although these analyses are not adjusted for age or other factors, a patient population of this size and breadth suggests the generalizability of the result.

The lack of a control group was a limitation of this study. Furthermore, many of the included registries were in early development at study initiation (in 2008) with less comprehensive data collection or fewer analysis options (e.g., comparator analyses) than are currently available. In addition, there was considerable variability among the registries in terms of design, determination of index date, completeness and manner of recording of concomitant treatments and known risk factors for serious infections or malignancies, data collection methods, computation of p-y exposed, definition/reporting of outcomes, validation of events, and calculation of IRs—all of which may have resulted in the wide range of IRs observed. This heterogeneity, as well as the possibility of under-reporting of adverse events in observational studies, in general, should be considered when assessing the potential clinical impact of these data.

## Conclusions

In this collaborative, international, real-world observational study using data from existing RA registries, no new or increased risks of infection or malignancy were identified for abatacept in the treatment of patients with RA. Despite the heterogeneity between registries, the data reported are consistent with the overall known safety profile for abatacept. Overall, most IRs were consistent with previously reported reference ranges.

## Supplementary Information

**Additional file 1.** Methods used to calculate patient-time of exposure and incidence rates for each individual registry included in the post-marketing epidemiology abatacept study.

## Data Availability

Bristol Myers Squibb policy on data sharing may be found at https://www.bms.com/researchers-and-partners/clinical-trials-and-research/disclosure-commitment.html.

## Abbreviations

ATTRA: Anti-TNF Therapy in Rheumatoid Arthritis
bDMARD: Biologic disease-modifying antirheumatic drug
BIOBADASER: Spanish Register of Adverse Events of Biological Therapies in Rheumatic Diseases
BSRBR: British Society for Rheumatology Biologics Register
CI: Confidence interval
csDMARD: Conventional synthetic disease-modifying antirheumatic drug
DANBIO: Danish Rheumatologic Database
DMARD: Disease-modifying antirheumatic drug
GISEA: Italian Group for the Study of Early Arthritis
HILMO: Finnish Nationwide Social and Healthcare Data Collection and Reporting System
ICD(-10): International Classification of Diseases (Tenth Edition)
IR: Incidence rate
MAH: Marketing authorization holder
MedDRA: Medical Dictionary for Drug Regulatory Activities
NR: Not reported
ORA: Orencia and Rheumatoid Arthritis
p-y: Patient-years
RA: Rheumatoid arthritis
ROB-FIN: National Registry of Antirheumatic and Biological Treatment in Finland
SCQM: Swiss Clinical Quality Management
SD: Standard deviation
TNF: Tumour necrosis factor
tsDMARD: Targeted synthetic disease-modifying antirheumatic drug
