# Supplementary material for: Safety outcomes in patients with rheumatoid arthritis treated with abatacept: results from a multinational surveillance study across seven European registries
Source: Arthritis Res Ther. 2023 Jun 12;25:101. doi: 10.1186/s13075-023-03067-x (PMC10259009; doi:10.1186/s13075-023-03067-x)
Supplement: Supplementary file 1 — Additional file 1. Methods used to calculate patient-time of exposure and incidence rates for each individual registry included in the post-marketing epidemiology abatacept study. [file 13075_2023_3067_MOESM1_ESM.docx]

**Additional file 1**

**Methods used to calculate patient-time of exposure and incidence rates (IRs) for each individual registry included in the post-marketing epidemiology abatacept study**

IRs with 95% confidence intervals (CIs; some registries used normal approximation, some used exact method) were provided for each prespecified outcome and reported to the Marketing Authorization Holder (MAH) by the registry, with the exception of GISEA (Italian Group for the Study of Early Arthritis). Variations in the methodology for the calculation of IRs for each registry are outlined below.

**ATTRA** (Anti-TNF Therapy in Rheumatoid Arthritis)

IRs were calculated as the number of events per 1000 total patient-years (p-y) of follow-up. IRs are followed by 95% Poisson CIs. Abatacept p-y were computed from the start of abatacept treatment to the end of abatacept follow-up (i.e. switch to another treatment, death, end of study, or withdrawal from the registry). Information about adverse events was gained using Medical Dictionary for Drug Regulatory Activities (MedDRA) system coding.

**DANBIO** (Danish Rheumatologic Database)

For infections, abatacept exposure was calculated from the date of treatment start (index date) until the event of interest, death, emigration, or end of follow-up (3 December 2017), whichever occurred first. IRs for infections were computed as number of events per 1000 exposed p-y of follow-up with associated 95% CIs. Patients contributed exposed follow-up time from date of first treatment of either tumor necrosis factor (TNF) inhibitor treatment or non-TNF biologic (b) disease-modifying antirheumatic drugs (DMARDs) or 15 June 2007 (whichever occurred latest) until 90 days after last treatment within the specific exposure group.

For malignancies, exposure was calculated as the total follow-up time up until the last follow-up date, end of the study, or death, whichever occurred first, but excluded the first 180 days from first exposure to abatacept (index date). IRs were computed as the number of events per 1000 total p-y of follow-up without inclusion of the first 180 days after first exposure to abatacept. Patients with previous diagnoses of any cancer (except non-melanoma skin cancer) were excluded when computing IRs. If the number of outcomes was >25, analyses were presented for patients receiving first-line treatment and switchers as well as combined therapy.

**ROB-FIN** (National Registry of Biological Treatment in Finland)

Infections requiring hospitalization were identified from the Care Register for Health Care. All hospitalizations due to a valid International Classification of Diseases, Tenth Edition (ICD-10) code (primary or secondary diagnosis) were included, but subsequent hospitalizations for the same ICD-10 diagnosis code were excluded. A single patient could contribute multiple events to the results. Malignancies were retrieved from the Finnish Cancer Registry.

Malignancies were captured by linkage to the Finnish nationwide social and healthcare data collection and reporting system (HILMO) and identified according to ICD-10 codes. For malignancies, time of exposure was the period from the beginning of therapy with abatacept relative to reporting of a malignancy event.

**ORA** (Orencia and Rheumatoid Arthritis)

Infection IRs were presented in two ways: the overall severe infection rate (“event rate”) accounting for the entirety of the follow-up period and the amount of infections for those within 3 months following infusion of abatacept (from the first date of infusion to the date of the last infusion + 3 months, or the last follow-up date). This latter method corresponds to the rate that was provided to the MAH in the final report from ORA. The ORA registry reports infections as severe infections. These infections require hospitalization, intravenous antibiotics, or are fatal.

Adverse event narrative information for malignancy cases was provided in the French ORA report. Follow-up for incidence of cancers is the overall follow-up period. No CIs were provided by the ORA registry for any of the incidence rates.

**GISEA** (Italian Group for the Study of Early Arthritis)

No IRs were provided by the registry. The MAH calculated the IRs using the mean duration of therapy (in months) taken from Table 8 of the November 2015 GISEA data report (Appendix 6) and the adverse event counts only for infections and neoplasms provided in the March 2014 GISEA report. The total number of abatacept users for first- and second-line therapy was used in the calculation of the p-y.

**BIOBADASER**

In the BIOBADASER registry, malignancies were identified according to MedDRA terms. For malignancies, abatacept patient-time at risk was the period from the start of abatacept treatment (time zero or index date) to the event of interest, to the date of the last administration plus twice the half-life of the drug, censor date (last visit in a patient lost to follow-up), death, or the end of observation period, whichever occurred first.

For infections, abatacept patient-time at risk is computed by the registry from the start of abatacept treatment (time zero or index date) to the event of interest, end of abatacept treatment, withdrawal from registry, death, or end of study. An additional sensitivity analysis of end of treatment + 90 days was performed. In this case, this window has considered for all groups except for malignancies that were considered “always at risk”.

**SCQM** (Swiss Clinical Quality Management)

Data collection periods differed for malignancies, tuberculosis, and hospitalized infections due to a lack of availability of certain data, particularly whether an infection had led to hospitalization.

With respect to infections, possible risk was attributable to the treatment immediately after initiation. For malignancies, the conveyed risks were considered everlasting to take into account a potentially delayed onset.

A key difference between the SCQM and other registries in this study is that the SCQM database lacks information on the exact date of occurrence for a proportion of the study’s endpoints. SCQM therefore imputed dates of occurrence using the limits of the time interval within which the event must have had occurred and reported the analyses on these imputed dates. Three types of imputation were utilized (left, mid, and right). Left and right identified both a date after which (left) and before which (right) the event took place. The midpoint between left and right imputed dates was the “mid” imputed date. For the purposes of this final study summary, the MAH summarized SCQM data by selecting the mid imputed dates.

The follow-up period was terminated as soon as a patient initiated treatment with DMARDs other than those under consideration. Patients for whom the follow-up period was undefined were excluded.

Definitions of follow-up for hospitalized infections and tuberculosis were as follows:

- The start of follow-up period was defined as either a) the latest of the start of study, first visit recorded in the registry, or last stop of bDMARD treatments when the start date was missing, or b) left undefined in cases where there were bDMARD treatments for which information on both the start and the stop dates was missing.
- In cases where abatacept had been started prior to and was ongoing at a patient’s first recorded visit, exposure was considered to be from the time of this first follow-up visit provided the patient had not experienced a hospitalized infection on a bDMARD treatment course before.
- A lag time of one standard dosing interval (e.g. 1 month for intravenously administered abatacept) was considered.
- The end of the follow-up period was defined either as a) the last visit recorded in the registry, an initiation of a new DMARD treatment other than those under consideration, or b) left undefined in case there were bDMARD treatments for which information on both the start and the stop date were missing.

Definitions of follow-up for malignancies were as follows:

- The start of the follow-up period was defined as either a) the first visit recorded in the registry or b) left undefined in cases there were DMARD treatments for which information on the start date was missing.
- The end of the follow-up period was defined as either a) the end of study, last visit recorded in the registry, and the initiation of treatments with DMARDs other than those under consideration or b) left undefined in case there were DMARD treatments for which information on the start date was missing.
